# EEG-based minimum spanning tree analysis reveals network disruptions in Alzheimer’s disease spectrum: an observational study

**DOI:** 10.3389/fnagi.2025.1604345

**Published:** 2025-09-26

**Authors:** Xing Ye, Yixin Yan, Yingying Wang, Jingping Shi

**Affiliations:** ^1^Department of Neurology, Nanjing Brain Hospital Affiliated to Nanjing Medical University, Nanjing, China; ^2^Department of Neurology, Shanghai Public Health Clinical Center, Fudan University, Shanghai, China; ^3^Department of Neurology, Huai'an 82 Hospital, Huai'an, Jiangsu, China

**Keywords:** Alzheimer’s disease, mild cognitive impairment, resting-state electroencephalogram, minimum spanning tree, Phase Lag Index

## Abstract

**Introduction:**

Alzheimer’s disease (AD) is characterized by disrupted brain connectivity, but the network changes across disease stages remain poorly understood. This observational cross-sectional study investigated alterations in functional brain networks across the AD continuum using minimum spanning tree (MST) analysis of resting-state EEG (rsEEG) data.

**Methods:**

We analyzed rsEEG data from 65 participants (30 healthy controls, 14 mild cognitive impairment due to AD [MCI-AD], 21 AD). Phase Lag Index (PLI)-based connectivity and MST metrics (such as diameter, eccentricity, and maximum degree) were computed across five frequency bands. Group differences were assessed using Kruskal-Wallis tests, and correlations with cognitive measures, disease severity, and cerebrospinal fluid (CSF) biomarkers were examined.

**Results:**

Significant alterations in rsEEG network topology were observed across HC, MCI-AD, and AD groups. AD patients showed increased theta band connectivity (higher mean PLI, diameter, and eccentricity) and decreased beta band connectivity (lower mean PLI and eccentricity) compared to HC. MCI-AD group exhibited higher delta band maximum degree and altered beta band network organization compared to HC and AD. These network changes correlated with cognitive performance and disease severity. Beta band mean PLI and theta band eccentricity effectively discriminated between AD/MCI-AD and HC. Significant correlations were also found between specific MST metrics and CSF biomarkers (t-Tau, p-Tau, Aβ_1–42_).

**Conclusion:**

AD progression is characterized by frequency-specific alterations in brain network topology, particularly in theta and beta bands, detectable through rsEEG-based MST analysis. These findings suggest EEG-derived network measures may serve as potential biomarkers for early AD diagnosis and monitoring disease progression.

## 1 Introduction

Alzheimer’s disease (AD) is a progressive neurodegenerative disorder characterized by severe memory impairment, cognitive decline, and a significant impact on quality of life (Gaugler et al., 2022). As the disease progresses, patients experience a decline in their ability to perform daily activities and maintain social relationships. The World Health Organization projects that AD will become a leading cause of disability and mortality among older adults by 2050 (Nichols et al., 2022; Scheltens et al., 2021). To better understand AD and develop effective treatments, it is essential to investigate its neurobiological signatures using multiple imaging modalities, including electroencephalography (EEG), magnetoencephalography (MEG), functional magnetic resonance imaging (fMRI), positron emission tomography (PET), and single-photon emission computed tomography (SPECT) (Albert et al., 2011; Davatzikos et al., 2011).

Recent studies have extensively explored AD using various approaches, including altered EEG spectral power, disrupted functional connectivity patterns, and changes in brain network topology (Perez-Valero et al., 2021; Scheijbeler et al., 2023; Kehm et al., 2023). These studies have reported increased slow-wave activity (delta and theta bands), decreased fast-wave activity (alpha and beta bands), reduced functional connectivity in higher frequency bands, and alterations in small-world network properties in AD patients. While these findings have significantly contributed to our understanding of AD, inconsistencies and contradictions exist due to differences in study populations, experimental designs, analytical methods, and other limitations (Maestú et al., 2015; Palop and Mucke, 2016; Engels et al., 2017; Horvath et al., 2018; Babiloni et al., 2020). Therefore, more advanced methods and techniques are needed to explore the complex nature of AD and its symptomatic predementia phase, mild cognitive impairment due to AD (MCI-AD), which is characterized by cognitive decline and may progress to AD-related dementia over time (Albert et al., 2011; Horvath et al., 2018; Meghdadi et al., 2021; Benwell et al., 2020; Tait et al., 2019).

Previous studies have shown that AD is associated with alterations in EEG spectral power and functional connectivity across different frequency bands. Graph theoretical approaches have significantly advanced the analysis of these complex EEG patterns (Youssef et al., 2021; Reijneveld et al., 2007). However, traditional methods of brain network analysis face several challenges. Conventional approaches rely on arbitrary thresholds to define network connections, which can lead to biases in network characterization. Furthermore, different thresholds used across studies hinder direct comparisons of network properties. The use of continuous association matrices in conventional neuroimaging techniques also presents a dense network of connections, which does not accurately reflect the brain’s sparse network structure (Bahrami et al., 2023; Bijsterbosch et al., 2020). This discrepancy complicates the accurate depiction of brain connectivity and introduces potential biases in subsequent analyses (Bahrami et al., 2023; Crimi et al., 2019).

To overcome the limitations of traditional network analysis methods, the Minimum Spanning Tree (MST) approach has emerged as a promising solution for studying brain networks in AD and other neurological disorders (Blomsma et al., 2022; Canario et al., 2022; Tewarie et al., 2015). MST is a subgraph that connects all nodes in the original weighted network without forming cycles, offering significant advantages over traditional approaches (Tewarie et al., 2015; Ciftçi, 2011). By retaining the most critical connections and preserving the network’s essential topological structure, MST ensures that all analyzed networks have the same number of nodes and links (Canario et al., 2022; Tewarie et al., 2015). This approach effectively captures the modular structure of brain networks, revealing areas with dense intra-module connections and sparse inter-module connections (Tewarie et al., 2015; Song et al., 2014; Becske et al., 2024; Tewarie et al., 2014). In AD research, MST’s ability to detect subtle changes in network topology is particularly valuable, as it can indicate compromised communication between brain regions and network disintegration into isolated clusters.

Recent studies, such as Canario et al. (2022), have shown that MST is effective in AD research, revealing less integrated network structures in AD patients compared to MCI and normal controls. This sensitivity to subtle changes in brain connectivity is crucial for understanding AD progression, especially during the early stages or the transition from MCI to AD. However, despite MST’s potential, previous EEG-based MST studies in AD have produced inconsistent results, with some studies reporting more line-like MST topology and others finding more centralized networks (Tijms et al., 2013). These discrepancies likely result from heterogeneous diagnostic criteria and the lack of biomarker confirmation, highlighting the need for more rigorous methodologies.

To address these limitations and clarify conflicting findings, our study uses a comprehensive diagnostic approach that combines established clinical criteria with advanced biomarker assessments. This approach ensures accurate patient classification and increases the reliability of our findings. By integrating resting-state EEG (rsEEG) data with neuropsychological tests and cerebrospinal fluid biomarkers, we aim to provide a more comprehensive understanding of AD-related network alterations. This multi-modal approach advances our understanding of AD and sets a new standard for the application of MST analysis in clinical neuroscience research.

Based on these considerations, we hypothesize that MST analysis of rsEEG data will reveal distinct network topologies that differentiate patients with MCI-AD, AD, and healthy controls, and correlate with clinical symptoms and cognitive impairment. We also expect that MST-derived metrics from rsEEG, such as leaf fraction, betweenness centrality, and tree hierarchy, will be significantly associated with neuropsychological test outcomes and cerebrospinal fluid biomarkers, including amyloid-beta (Aβ) and tau proteins. This approach aims to clarify conflicting findings and identify more reliable EEG-derived network biomarkers for AD, providing consistent insights into the neuropathology of AD and improving early detection and disease monitoring.

## 2 Method

### 2.1 Participants

We conducted our study with a cohort comprising 14 individuals diagnosed with MCI-AD, 21 individuals with AD, and 30 HC participants, recruited from the Neurology Department of Nanjing Brain Hospital between October 2020 and May 2022. The participants were diagnosed using standard clinical criteria and biomarker detection, including Aβ deposition and tau pathology, through cerebrospinal fluid analysis, following the NIA-AA research framework guidelines (Jack et al., 2018).

The inclusion criteria for all participants were: right-handed adults aged 50–79 years, no significant visual or auditory impairments, and a minimum of 6 years of education. Furthermore, HC participants were required to perform daily activities independently, while MCI-AD participants required memory concerns substantiated by MMSE scores > 20 and meeting the Alzheimer’s continuum criteria. AD participants required a clinical AD diagnosis with significant cognitive decline and MMSE scores <20.

Exclusion criteria included participants with cognitive decline due to other conditions, major psychiatric disorders, substance abuse, or other neurological disorders affecting cognitive function. These standardized criteria ensured a homogeneous sample, minimizing confounding variables.

The control group consisted of healthy elderly adults who were community volunteers, with no evidence of dementing or other neuropsychological disorders. The study was approved by the Medical Research Ethics Committee of the Brain Hospital Affiliated to Nanjing Medical University. Informed consent was obtained from all participants prior to the initiation of the study.

### 2.2 Clinical and neuropsychological assessments

Participants in both groups underwent comprehensive and standard clinical and neuropsychological evaluations. The test battery included the MMSE and the Montreal Cognitive Assessment (MoCA) for assessing global cognitive function (Horton et al., 2015; Tombaugh and McIntyre, 1992); the Revised Hasegawa’s Dementia Scale (HDS-R) for assessing the severity of dementia (Imai and Hasegawa, 1994); the Clinical Dementia Rating (CDR) for evaluating cognitive and social functioning (Morris, 1993); the Auditory Verbal Learning Test (AVLT) for assessing verbal memory and learning (Vakil and Blachstein, 1993); and the Hamilton Anxiety Scale (HAMA) and Hamilton Depression Scale (HAMD) (Maier et al., 1988; O'Hara and Rehm, 1983) for evaluating emotional state. Experienced neuropsychologists assessed participants’ general cognitive function, episodic memory, and emotional state using these scales.

### 2.3 Analysis of cerebrospinal fluid markers

All participants in the MCI-AD and AD groups underwent lumbar punctures to confirm the cerebrospinal fluid (CSF) profile indicative of AD pathology. This included measuring the concentrations for Aβ_1–42_ and Aβ_1–40_, the ratio of Aβ_42_/Aβ_40_, and the levels of phosphorylated tau (p-Tau) and total tau proteins (t-Tau). The INNO-BIA AlzBio3 immunoassay kit (Innotest, Fujirebio, Ghent, Belgium) was utilized for these determinations. Threshold values were established based on prior research and insights from our laboratory (Mulder et al., 2010).

### 2.4 EEG data acquisition

EEG recordings were captured using 64 Ag/AgCl electrodes placed on a BrainCap elastic cap according to the international 10–20 system. Electrodes for horizontal and vertical eye movements were positioned at the outer canthus of the right eye and above the inner canthus of the left eye, respectively. The EEG signals were recorded at a sampling rate of 1,000 Hz using the BrainAmp DC amplifier (Brain Products GmbH, Gilching, Germany). All electrode impedances were maintained below 10kΩ. EEG recordings took place between 10 a.m. and 4 p.m. on working days. Participants sat in a dimly lit and quiet room, remaining alert and relaxed, and underwent an 8 min eyes-closed recording session.

### 2.5 EEG data preprocessing

EEG data were preprocessed using EEGLAB v.13.5.4 (Delorme and Makeig, 2004) on the Matlab2021a platform (Mathworks, Inc., Natick, MA, United States) following these steps: (1) EOG channels were excluded from the analysis; (2) Raw EEG signals were re-referenced offline to the global cerebral average reference; (3) The data were then band-pass filtered offline between 0.5–48 Hz; (4) The filtered data were down-sampled to 250 Hz; (5) The data were segmented into 2 s epochs; (6) Epochs containing artifacts such as eye movements, muscle activity, and line noise were visually inspected and excluded; and (7) A minimum of 40 artifact-free 2 s epochs per participant were included in the analysis. The functional connectivity analysis and subsequent brain network topology analysis were conducted using the FieldTrip toolbox (Oostenveld et al., 2011).

### 2.6 Functional connectivity analysis

Functional connectivity is assessed using the Phase Lag Index (PLI), a robust method that quantifies the asymmetry in the distribution of phase differences between two signals (Stam et al., 2007). The PLI quantifies the asymmetry of phase difference distributions between two signals, offering robustness against volume conduction effects. For time series X and Y with phase difference Δφ(t), the PLI is defined as:


PLI=∣〈sign[sin(Δφ(t))]〉∣

PLI values range from 0 (no coupling) to 1 (perfect phase locking). Increased PLI values suggest enhanced phase locking between two signals. We computed PLI between all pairs of 62 EEG channels across five frequency bands: Delta (0.5–4 Hz), Theta (4–8 Hz), Alpha (8–13 Hz), Beta (13–30 Hz), and Gamma (30–48 Hz). The mean PLI across all channel pairs in each band was calculated to represent global brain synchronization. We chose PLI over its variants, such as the Weighted Phase Lag Index (wPLI), for two primary reasons. First, this approach preserves methodological continuity with prior EEG-MST studies in Alzheimer’s Disease. Second, it avoids the complexities of parameter tuning (e.g., squared or debiased variants), which can be particularly challenging with short clinical rsEEG segments. Notably, our choice is further justified by our use of MST, a threshold-free method that relies on the relative ranking of edge weights (1 − PLI), ensuring stable topological comparisons across our heterogeneous clinical population.

### 2.7 Minimum spanning tree analysis

In this section, we describe the construction of the MST for each frequency band using a 62×62 PLI adjacency matrix. To prepare the PLI values for MST analysis, we transform the PLI matrix into a weight matrix by subtracting the PLI values from 1, such that lower values in the resulting matrix indicate stronger connections due to higher phase synchrony. Subsequently, we employ the Kruskal algorithm to construct the MST (Kruskal, 1956). This algorithm involves sorting all connections according to their weights in ascending order, where smaller values represent stronger and more critical connections. Each connection, starting with the one with the smallest weight, is added to the MST only if it does not form a cycle with the connections already included. Connections that would lead to a cycle are excluded. This procedure is repeated until a spanning tree is formed that connects all N nodes (where N = 62, representing the EEG channels) with M = N − 1 edges (i.e., 61 edges), thereby capturing all essential connections based on the strongest phase synchrony.

#### 2.7.1 Metrics for comparing MST topological structures

Several metrics are employed to compare MST topological structures, including Degree_max_, Kappa, Betweenness Centrality (BC), Diameter, Eccentricity (Ecc), Leaf fraction (Lf), and tree hierarchy (Th) (Tewarie et al., 2015; Stam et al., 2014). Table 1 summarizes the definitions and interpretations of all MST metrics utilized in this study. These metrics include node-specific measures (Degree, BC, Eccentricity), their tree-level aggregates (Degree_max_, BC_max_, Ecc_mean_), and global tree characteristics (e.g., Kappa, Diameter, Leaf fraction, Tree hierarchy).

**Table 1:** Descriptive parameters of MST features.

Symbol	Feature	Explanation	Mathematical expression
N	Nodes	Number of nodes in the MST.	–
M	Links	Number of connections in the MST.	–
K	Degree	Number of connections passing through a node. ${\text{Degree}}_{\max}$ denotes the maximum degree among all nodes, indicative of a “hub” or key area in functional brain networks.	$\mathrm{Ki}=\sum_{j=1,j\neq i}^{N}e$*ij*
BC	Betweenness centrality	Probability of a node on shortest paths between other nodes. ${\mathrm{BC}}_{\max}$ is the maximum BC value among all nodes in the MST, describing the importance of central node and network center efficiency.	${\mathrm{BC}}_{i}=\frac{1}{\left(N-1)(N-2\right)}\sum_{h,j\in N,h\neq i,h\neq j,i\neq j}\frac{\rho_{\mathrm{ij}}(i)}{\rho_{\mathrm{ij}}}$
Ecc	Eccentricity	The longest shortest path from a node to any other node in the MST. Low average eccentricity implies a more centralized node status in the MST.	$E_{i}=\max\{d(i,j)|j\in N\}$
D	Diameter	Indicates the overall network organization efficiency, measured by the maximum path length in the MST. A smaller network diameter indicates more effective information processing between distant brain regions.	D=d/M
Lf	Leaf fraction	Measured by the number of leaves (nodes with only one connection) in the MST.	$L_{f}=L∕M$
Th	Tree hierarchy	Quantifies the balance between large-scale concentration and central node load in the MST.	$T_{h}=\frac{L}{2\text{MBCmax}}$
k	Kappa	Measures the breadth of degree distribution. A network with a lower score is more vulnerable to attacks, potentially leading to more significant damage to overall network functionality.	$k=\frac{k^{2}}{k}$

This table summarizes the definitions and interpretations of all MST features used in this study. Definitions and notions adapted from the studies by Tewarie et al. (2015) and van Dellen et al. (2014). $\rho_{\mathrm{ij}}$ is the number of shortest paths between nodes i and j, and $\rho_{\mathrm{ij}}(i)$ is the number of shortest paths between i and j that pass through i.$\;d_{\mathrm{ij}}$represents the shortest path between nodes i and j. Lf denotes the total number of leaves in the MST.

#### 2.7.2 Topological extremes of MST

**Figure 1:**
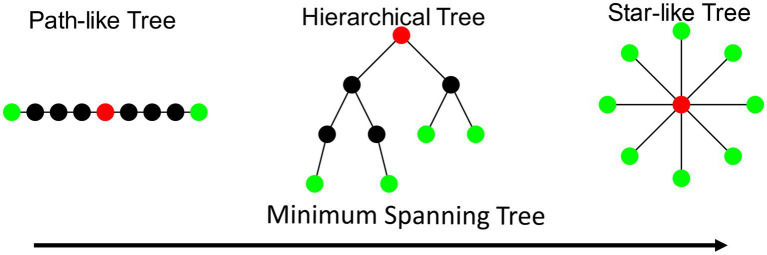
Topological variants of the MST. Three distinct configurations of MSTs are illustrated: path-like tree (left), characterized by a linear arrangement of nodes; hierarchical tree (center), showing a branching structure with multiple levels; and star-like tree (right), featuring a central hub node connected to multiple peripheral nodes. Red nodes indicate central or key positions in each topology, while green nodes represent leaf or terminal positions.

In MST analysis, two topological extremes are identified: the chain and star shapes (Figure 1). The chain form is characterized by an elongated structure where all nodes connect to the endpoints, resulting in lower Degree_max_ and BC_max_, a longer diameter, and fewer leaf nodes. Conversely, a star-shaped MST features a central node connected to all other nodes, exhibiting higher Degree_max_ and BC_max_ values, a shorter diameter, and more leaf nodes. While the star-shaped topology is highly efficient for information transmission, it is vulnerable to overload due to its centralized structure. The optimal MST topology lies between these two extremes, balancing central concentration and node load (van Dellen et al., 2014; van Lutterveld et al., 2017). This balance is quantified through the Tree hierarchy metric, which measures the degree of equilibrium in the tree structure.

#### 2.7.3 EEG data processing and network analysis workflow

**Figure 2:**
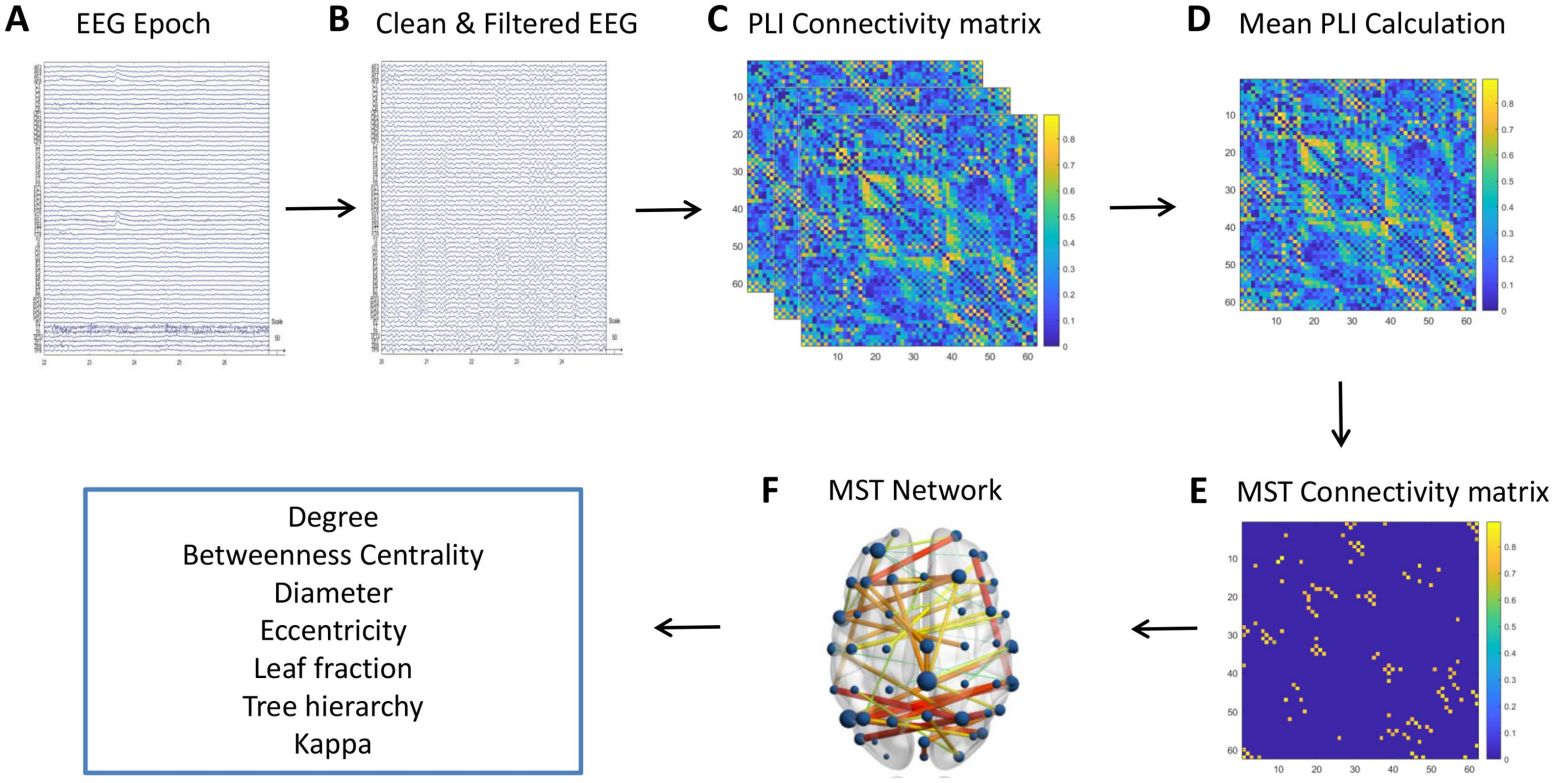
Workflow for processing EEG data to analyze brain connectivity. **(A)** Raw EEG epochs. **(B)** Cleaned and filtered EEG data, with artifacts removed. **(C)** PLI connectivity matrix illustrating synchronization between EEG channels. **(D)** Mean PLI calculation providing a summary of overall brain connectivity. **(E)** MST connectivity matrix representing the backbone structure of the network, with color-coded connection strengths. **(F)** 3D visualization of the MST network on a brain model, highlighting the spatial distribution of brain connectivity.

Figure 2 illustrates the step-by-step workflow for processing and analyzing EEG data to study brain connectivity. Starting with raw EEG epochs (Panel A), artifacts are removed from the data, followed by band-pass filtering to remove noise (Panel B). Subsequently, the PLI connectivity matrix is computed to evaluate synchronization between EEG channels (Panel C). This matrix is then used to calculate the mean PLI, which provides a summary of overall brain connectivity (Panel D). Using this data, a MST connectivity matrix is generated (Panel E), which simplifies the network by focusing on the most significant connections. Finally, the resulting MST is visualized on a 3D brain model (Panel F), highlighting important network properties such as leaf fraction, betweenness centrality, and tree hierarchy, and providing insights into the functional architecture of the brain’s connectivity network.

### 2.8 Statistical methods

Statistical analyses were conducted using SPSS version 19.0 (SPSS Inc., Chicago, Illinois, United States) to analyze the demographic, clinical data, biomarkers, and the correlation with MST features of the subjects. Data is represented as mean ± standard deviation (SD). For normally distributed data, one-way ANOVA was used for multiple group comparisons, followed by LSD-t test for pairwise comparison. For non-normally distributed data, the Mann–Whitney U test was used for two-group comparison, and the Kruskal-Wallis test was used for multiple group comparisons, followed by Dunn’s test for further pairwise comparisons. To control for multiple testing, the Holm-Bonferroni method was used to adjust *p*-values. Categorical data were analyzed with a Chi-square test. All tests were two-tailed, and statistical significance was defined as *p* < 0.05.

## 3 Results

### 3.1 Demographic and clinical data of participants

This study included 30 healthy controls, 14 MCI-AD patients, and 21 AD patients. The sample size was determined based on the availability of eligible participants within our recruitment period and resources, while aiming to maintain group sizes comparable to previous EEG studies in Alzheimer’s disease. No significant differences were observed among the three groups in terms of gender distribution (*p* = 0.364), age (*p* = 0.336), and years of education (*p* = 0.062), providing a homogeneous baseline for subsequent cognitive comparisons.

#### 3.1.1 Cognitive function assessments

The AD group exhibited significantly lower cognitive function compared to both the HC and MCI-AD groups, as assessed by the MMSE and MoCA (Table 2). The mean MMSE scores were 28.16 ± 1.34, 26.40 ± 1.74, and 14.89 ± 5.91 for the HC, MCI-AD, and AD groups, respectively (*p* < 0.001). Similarly, the mean MoCA scores were 26.60 ± 1.81, 20.40 ± 4.45, and 9.26 ± 5.59 for the HC, MCI-AD, and AD groups, respectively (*p* < 0.001). The severity of dementia, as measured by the CDR and HDS-R, was significantly higher in the AD group compared to the HC group. The mean CDR scores were 0.00 ± 0.00 and 1.05 ± 0.62 for the HC and AD groups, respectively (*p* < 0.001). The mean HDS-R scores were 31.15 ± 1.46 and 13.04 ± 9.27 for the HC and AD groups, respectively (*p* < 0.001). The AVLT Delayed recall scores significantly differed among the groups (*p* < 0.001, Kruskal-Wallis Test), with the HC group scoring the highest (5.70 ± 2.67), followed by the MCI-AD group (1.86 ± 2.12) and the AD group (0.13 ± 0.52). Similarly, the AVLT Recognition scores were highest in the HC group (22.27 ± 1.72), lower in the MCI-AD group (17.14 ± 4.67), and lowest in the AD group (11.58 ± 8.79) (*p* < 0.001, Kruskal-Wallis Test).

**Table 2:** Demographic and clinical data of subjects.

Variable	HC (*n* = 30)	MCI-AD (*n* = 14)	AD (*n* = 21)	*p* value	*Post hoc*
Gender (*n*, % female)	18 (60.00%)	7 (46.67%)	14 (63.64%)	0.364	b
Age (years, M ± SD)	64.13 ± 8.18	67.20 ± 9.17	63.05 ± 8.43	0.336	a
Education (years, M ± SD)	13.30 ± 3.15	11.93 ± 3.71	10.65 ± 4.42	0.062	c
MMSE (M ± SD)	28.16 ± 1.34^AD^	26.40 ± 1.74^AD^	14.89 ± 5.91^HC, MCI-AD^	<0.001	a
MoCA (M ± SD)	26.60 ± 1.81^MCI-AD, AD^	20.40 ± 4.45^HC, AD^	9.26 ± 5.59^HC, MCI-AD^	<0.001	a
HDS-R (M ± SD)	31.15 ± 1.46 ^AD^	28.57 ± 3.19 ^AD^	13.04 ± 9.27 ^HC, MCI-AD^	<0.001	a
CDR (M ± SD)	0.00 ± 0.00 ^MCI-AD, AD^	0.33 ± 0.24 ^HC, AD^	1.05 ± 0.62 ^HC, MCI-AD^	<0.001	c
AVLT Delayed (M ± SD)	5.70 ± 2.67 ^AD^	1.86 ± 2.12 ^AD^	0.13 ± 0.52 ^HC, MCI-AD^	<0.001	c
AVLT Recognition (M ± SD)	22.27 ± 1.72 ^MCI-AD, AD^	17.14 ± 4.67 ^HC^	11.58 ± 8.79 ^HC^	<0.001	c
Aβ_1–42_ (pg/ml, M ± SD)	–	417.30 ± 131.76	449.22 ± 141.54	0.327	d
Aβ_1–40_ (pg/ml, M ± SD)	–	8641.30 ± 3744.81	10,386 ± 4835.20	0.304	d
Aβ_1–42_/Aβ_1–40_ Ratio	–	0.05 ± 0.16	0.048 ± 0.18	0.642	d
t-Tau (pg/ml, M ± SD)	–	566.37 ± 361.19	719.31 ± 309.02	0.139	d
p-Tau (pg/ml, M ± SD)	–	74.32 ± 73.16	121.03 ± 136.18	0.133	d

Superscripts denote significant between-group differences (*p* < 0.05). Statistical tests: a, ANOVA with post hoc Tukey’s test; b, Chi-Square test; c, Kruskal-Wallis test; d, Mann–Whitney U test. “–” indicates data not collected for HC group. t-Tau, total tau; p-Tau, phosphorylated tau.

#### 3.1.2 CSF biomarkers

No significant differences were observed between the MCI-AD and AD groups in the levels of Aβ_1–42_, Aβ_1–40_, and the Aβ_1–42_/Aβ_1–40_ ratio (Table 2). The mean Aβ_1–42_ levels were 417.30 ± 131.76 pg./mL and 449.22 ± 141.54 pg./mL, while the mean Aβ_1–40_ levels were 8641.30 ± 3744.81 pg./mL and 10,386 ± 4835.20 pg./mL for the MCI-AD and AD groups, respectively. The Aβ_1–42_/Aβ_1–40_ ratio was 0.05 ± 0.16 and 0.48 ± 0.18 for the MCI-AD and AD groups, respectively. Similarly, no significant differences were found between the MCI-AD and AD groups for t-Tau and p-Tau levels. The mean t-Tau levels were 566.37 ± 361.19 pg./mL and 719.31 ± 309.02 pg./mL, while the mean p-Tau levels were 74.32 ± 73.16 pg./mL and 121.03 ± 136.18 pg./mL for the MCI-AD and AD groups, respectively.

### 3.2 Inter-group analysis of whole-brain average PLI and MST features across frequency bands

#### 3.2.1 Whole-brain average PLI

**Figure 3:**
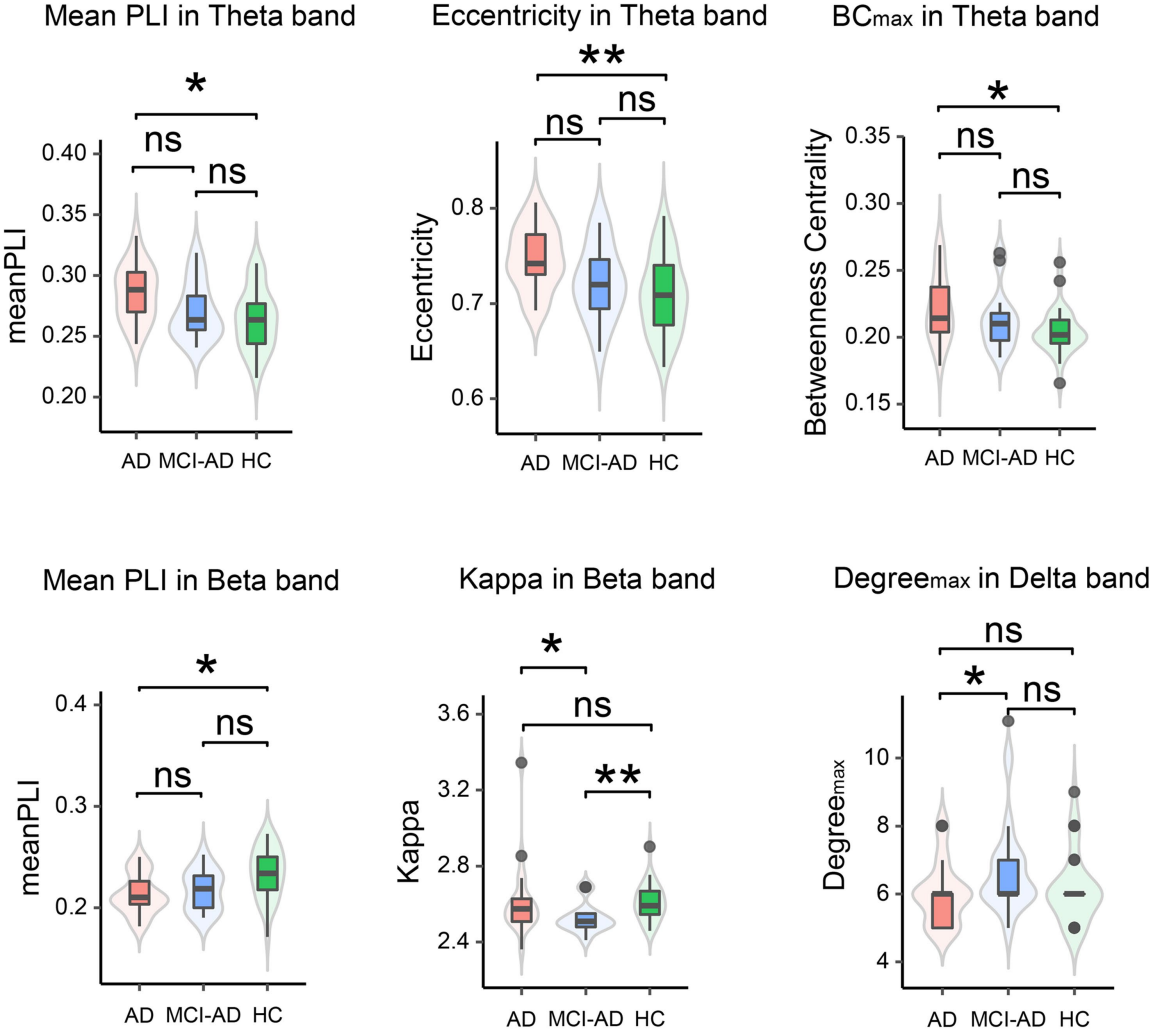
Violin plots representing inter-group comparisons of whole-brain PLI and MST features across different frequency bands. In each boxplot, the central line represents the median; the lower and upper edges of the box indicate the 25th and 75th percentiles, respectively; and the whiskers extend to 1.5 times the interquartile range. Overlaid violin plots show the probability density of the data. Statistical significance between groups is denoted as: * *p* < 0.05, ** *p* < 0.01, ns: not significant.

The Kruskal-Wallis test was applied to compare the mean PLI values for each frequency band, followed by Dunn’s test for pairwise comparisons. The AD group showed a significant increase in mean PLI within the Theta frequency band (χ^2^(2) = 8.11, *p* = 0.017) with a value of 0.285 ± 0.023, and a significant decrease in the Beta frequency band (χ^2^(2) = 9.94, *p* = 0.007) with a value of 0.213 ± 0.020 compared to the HC group. Interestingly, the MCI-AD group did not display significant differences when compared to both the HC and AD groups (refer to Table 3; Figure 3).

**Table 3:** Comparative analysis of network metrics across frequency bands for HC, MCI-AD, and AD groups.

Metric		AD (*N* = 21)		MCI-AD (*N* = 14)		HC (*N* = 30)		Kruskal-Wallis Test χ^2^ *(p)*	Dunn’s test (*p*) for multiple comparison
		M	(SD)	M	(SD)	M	(SD)		
Delta	Mean PLI	0.254	(0.029)	0.253	(0.033)	0.243	(0.044)	4.30 (0.117)	
	BC_max_	0.207	(0.029)	0.194	(0.024)	0.197	(0.022)	1.48 (0.478)	
	Diameter	0.865	(0.042)	0.867	(0.051)	0.845	(0.053)	4.13 (0.127)	
	Eccentricity	0.699	(0.038)	0.693	(0.044)	0.677	(0.052)	4.63 (0.099)	
	Degree_max_	**6.000**	**(0.918)**	**6.643**	**(1.216)**	**6.133**	**(1.008)**	**8.30 (0.016)**	MCI-AD vs. HC (0.018)
	Lf	0.478	(0.035)	0.478	(0.038)	0.455	(0.054)	2.48 (0.290)	
	Th	0.370	(0.036)	0.373	(0.036)	0.353	(0.050)	1.48 (0.478)	
	Kappa	2.726	(0.128)	2.787	(0.157)	2.698	(0.147)	0.84 (0.656)	
Theta	Mean PLI	**0.285**	**(0.023)**	**0.270**	**(0.024)**	**0.263**	**(0.025)**	**8.11 (0.017)**	AD vs. HC (0.014)
	BC_max_	**0.222**	**(0.025)**	**0.213**	**(0.023)**	**0.204**	**(0.017)**	**7.71 (0.021)**	AD vs. HC (0.018)
	Diameter	*0.873*	*(0.044)*	*0.866*	*(0.043)*	*0.851*	*(0.040)*	*5.98 (0.053)*	
	Eccentricity	**0.747**	**(0.033)**	**0.722**	**(0.039)**	**0.709**	**(0.041)**	**10.70 (0.005)**	AD vs. HC (0.003)
	Degree_max_	5.500	(1.000)	5.643	(0.745)	5.700	(1.557)	1.09 (0.580)	
	Lf	0.436	(0.022)	0.439	(0.033)	0.439	(0.037)	0.55 (0.758)	
	Th	0.361	(0.029)	0.352	(0.029)	0.347	(0.031)	1.91 (0.385)	
	Kappa	2.593	(0.085)	2.604	(0.081)	2.618	(0.192)	0.33 (0.846)	
Alpha	Mean PLI	0.303	(0.047)	0.301	(0.039)	0.312	(0.056)	0.50 (0.779)	
	BC_max_	0.200	(0.029)	0.216	(0.026)	0.207	(0.022)	3.19 (0.203)	
	Diameter	0.877	(0.033)	0.885	(0.034)	0.879	(0.046)	0.62 (0.733)	
	Eccentricity	0.759	(0.043)	0.760	(0.041)	0.767	(0.060)	0.23 (0.891)	
	Degree_max_	6.250	(1.943)	5.571	(0.756)	6.233	(1.547)	1.40 (0.497)	
	Lf	0.460	(0.044)	0.434	(0.040)	0.456	(0.036)	3.13 (0.209)	
	Th	0.361	(0.041)	0.351	(0.039)	0.356	(0.036)	0.25 (0.882)	
	Kappa	2.706	(0.231)	2.596	(0.104)	2.682	(0.157)	2.22 (0.329)	
Beta	Mean PLI	**0.213**	**(0.020)**	**0.217**	**(0.020)**	**0.234**	**(0.025)**	**9.94 (0.007)**	AD vs. HC (0.011)
	BC_max_	0.194	(0.016)	0.205	(0.026)	0.197	(0.026)	1.50 (0.473)	
	Diameter	0.840	(0.045)	0.812	(0.048)	0.829	(0.049)	3.53 (0.171)	
	Eccentricity	*0.661*	*(0.022)*	*0.668*	*(0.016)*	*0.680*	*(0.032)*	*6.43 (0.040)*	
	Degree_max_	*5.900*	*(1.294)*	*5.286*	*(0.611)*	*5.667*	*(1.028)*	*5.75 (0.056)*	
	Lf	*0.432*	*(0.043)*	*0.404*	*(0.031)*	*0.437*	*(0.036)*	*7.38 (0.025)*	
	Th	0.340	(0.033)	0.337	(0.029)	0.342	(0.038)	0.41 (0.814)	
	Kappa	**2.617**	**(0.202)**	**2.526**	**(0.079)**	**2.609**	**(0.098)**	**13.52 (0.001)**	MCI-AD vs. AD (0.046), MCI-AD vs. HC (0.001)
Gamma	Mean PLI	*0.188*	*(0.022)*	*0.189*	*(0.025)*	*0.199*	*(0.021)*	*5.54 (0.063)*	
	BC_max_	0.201	(0.020)	0.199	(0.023)	0.204	(0.020)	0.43 (0.809)	
	Diameter	0.867	(0.054)	0.845	(0.070)	0.840	(0.065)	2.18 (0.336)	
	Eccentricity	0.641	(0.021)	0.644	(0.029)	0.642	(0.029)	0.48 (0.785)	
	Degree_max_	6.250	(2.291)	5.500	(0.855)	5.933	(1.617)	0.77 (0.680)	
	Lf	0.444	(0.061)	0.426	(0.042)	0.441	(0.056)	0.82 (0.662)	
	Th	0.350	(0.055)	0.343	(0.038)	0.345	(0.053)	0.12 (0.941)	
	Kappa	2.688	(0.300)	2.576	(0.113)	2.649	(0.203)	0.89 (0.640)	

Values in bold indicate statistical significance (*p* < 0.05). Italicized values denote trend-level significance (0.05 ≤ *p* < 0.10). MST, minimum spanning tree; PLI, Phase Lag Index; BC, betweenness centrality; Th, tree hierarchy.

#### 3.2.2 MST attributes

Using the same statistical approach as for the whole-brain average PLI, we analyzed the MST features, with the addition of Holm-Bonferroni correction for multiple comparisons. This analysis revealed frequency-specific differences in brain network parameters across AD, MCI-AD, and HC groups. In the delta band, Max degree showed significant group differences (χ2 = 8.30, *p* = 0.016), with MCI-AD exhibiting higher values than HC (*p* = 0.018). The theta band demonstrated significant group effects in BC_max_ (χ2 = 7.71, *p* = 0.021) and Eccentricity (χ2 = 10.70, *p* = 0.005), with AD consistently showing higher values compared to HC (all *p* < 0.05). No significant differences were observed in the alpha band. In the beta band, Kappa showed significant differences (χ2 = 13.52, *p* = 0.001), with MCI-AD differing from both AD (*p* = 0.046) and HC (*p* = 0.001). Eccentricity (χ2 = 6.43, *p* = 0.040) and Leaf fraction (χ2 = 7.38, *p* = 0.025) in the beta band showed significant group effects, but post-hoc tests did not reach statistical significance. The gamma band showed no significant group differences in MST attributes.

### 3.3 Visualization and analysis of frequency-specific MST brain network connectivity

**Figure 4:**
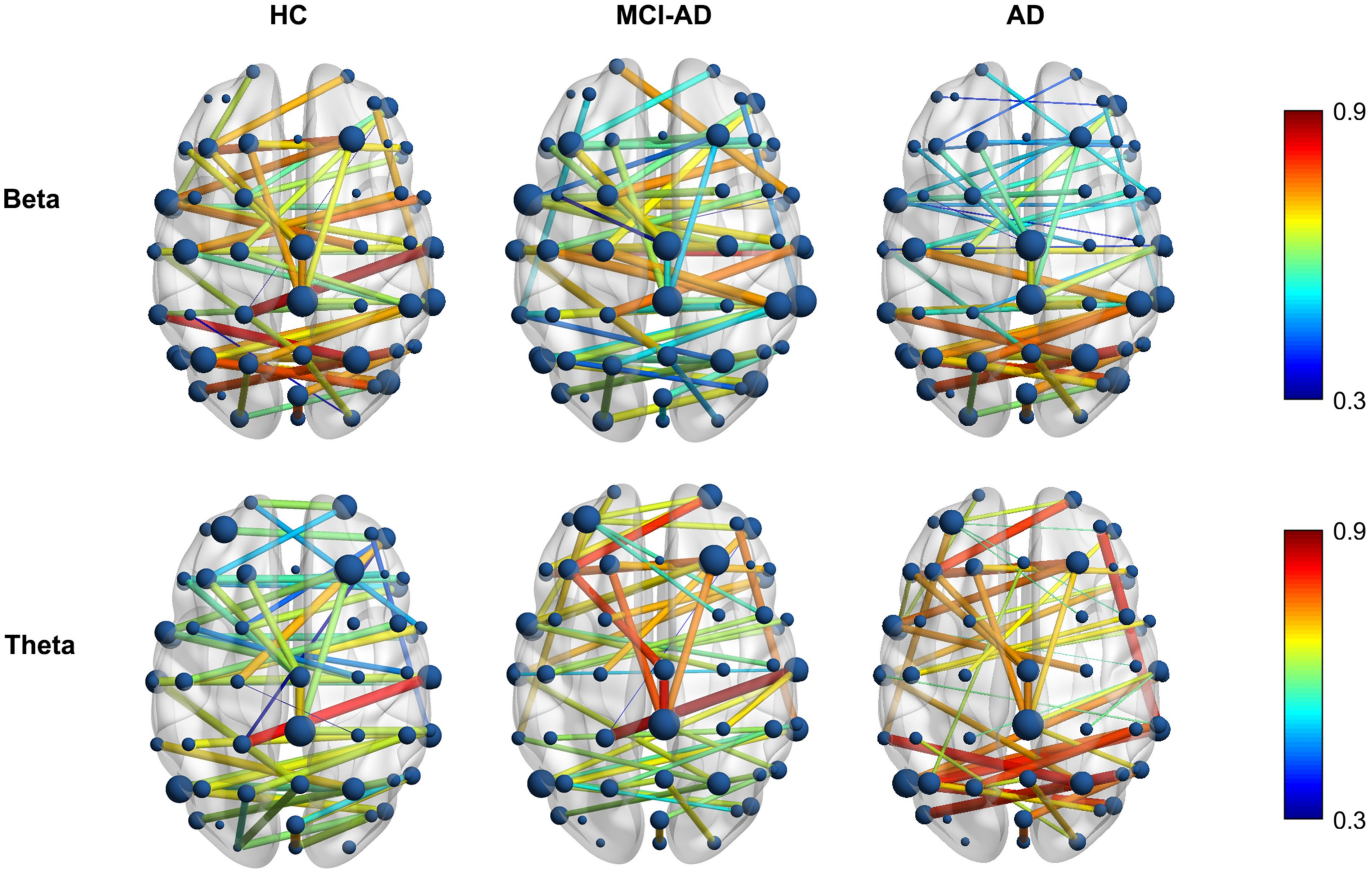
Differential distribution of MST brain network alterations in AD and MCI-AD. Topographical distribution of mean PLI values in MST brain networks for beta (13–30 Hz, top row) and theta (4–8 Hz, bottom row) frequency bands across HC, MCI-AD, and AD groups. The color scale represents PLI values from 0.3 (blue) to 0.9 (red).

This section extends our previous findings by visualizing and analyzing the functional connectivity patterns in the beta and theta frequency bands using MST brain network maps (Figure 4). These bands were selected based on their significant inter-group differences in PLI values and MST features, as highlighted in our earlier analyses. The brain connectivity maps provide a spatial representation of the network changes, offering insights into the regional specificity of alterations in functional connectivity across different stages of cognitive decline.

#### 3.3.1 Beta band connectivity patterns

In the beta band, the HC group displayed a well-distributed network with strong connections, particularly in the posterior and central regions. The MCI-AD group showed a marked reduction in connection strength and more dispersed network patterns, while the AD group demonstrated a further decline, with sparse and weaker connections primarily in the central and frontal regions. Notably, the AD group exhibited a more pronounced decline in functional connectivity within the anterior regions compared to the posterior regions, highlighting the varying effects of Alzheimer’s on different brain areas. Overall, the beta band exhibits a gradual decrease in PLI values from HC to AD, indicating a disruption of network integrity.

#### 3.3.2 Theta band connectivity patterns

In contrast to the beta band, the theta band shows a progressive increase in PLI values from HC to AD. The HC group exhibited a robust network with strong connections distributed across the brain, signifying a balanced and widespread interregional interplay. The MCI-AD group displayed a decrease in connectivity strength, especially in the posterior regions, although some residual high-weight edges persisted. The AD group showed the most pronounced changes, with an overall sparser network, and a relative posterior predominance of the remaining high-weight edges, indicating a spatial shift of connectivity despite reduced global density.

### 3.4 Correlation analysis of clinical features and MST network measures

**Figure 5:**
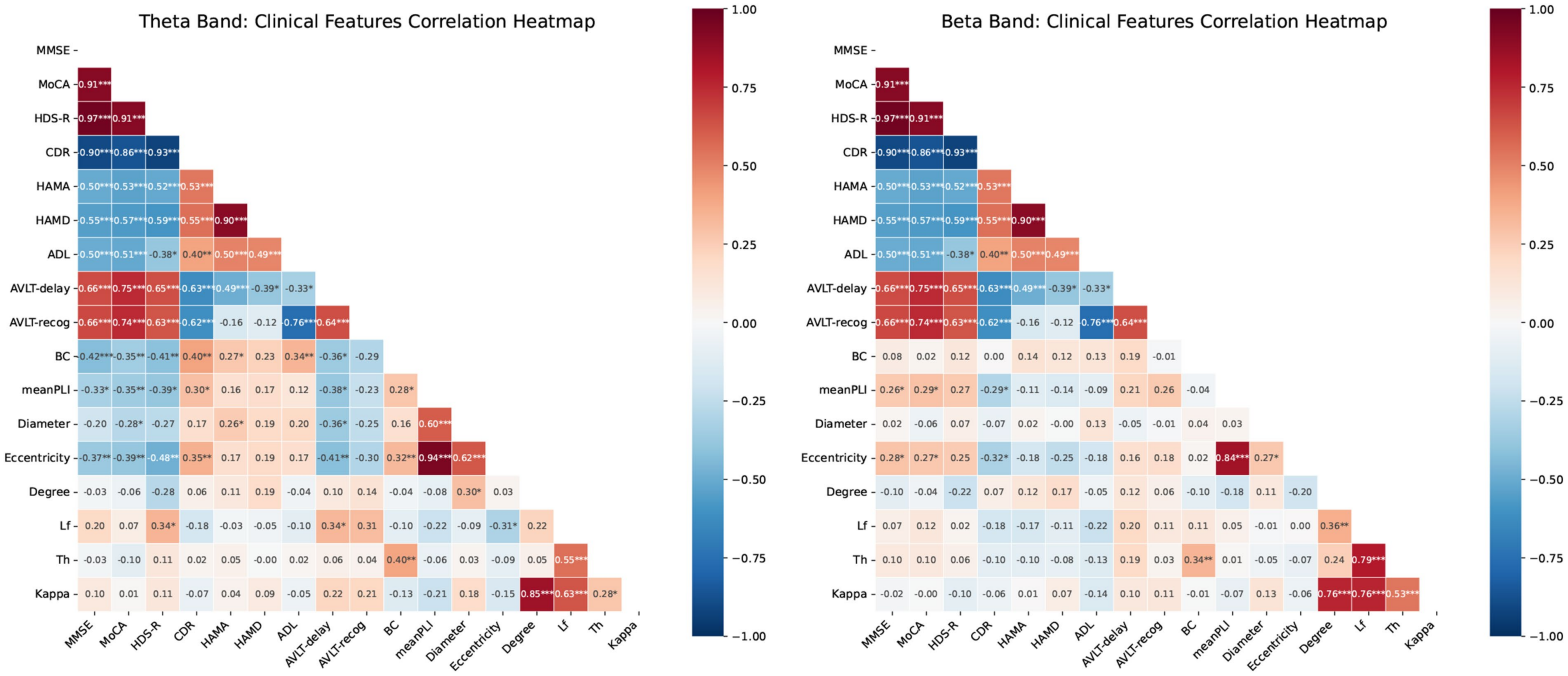
Correlation heatmaps for MST features and cognitive scores. **(A)** Theta band: Pearson correlations between network metrics (PLI, BC, eccentricity, Lf, degree, diameter, Th) and clinical scores (MMSE, MoCA, HDS-R, CDR, HAMA, HAMD, ADL, AVLT-delay, AVLT-recog). **(B)** Beta band: correlations between identical metrics and scores in the beta frequency range. Blue and red indicate positive and negative correlations, respectively. Color intensity represents the Pearson correlation coefficient (−1 to 1). Significance: * *p* < 0.05, ** *p* < 0.01, ***p < 0.001. mean PLI, mean Phase Lag Index; BC, betweenness centrality; Th, tree hierarchy; Lf, leaf fraction.

Correlation analysis revealed significant associations between MST network metrics and clinical scores, with distinct patterns across frequency bands (Figure 5). In the theta band, key metrics such as Mean PLI, BC, and Eccentricity were robustly correlated with poorer cognitive performance (e.g., negative correlations with MMSE and MoCA) and greater disease severity (positive correlation with CDR). In contrast, the beta band showed an opposing trend, where Mean PLI and Eccentricity were significantly associated with better cognitive outcomes and lower disease severity.

### 3.5 ROC analysis of MST features for differentiating AD, MCI-AD, and HC

**Figure 6:**
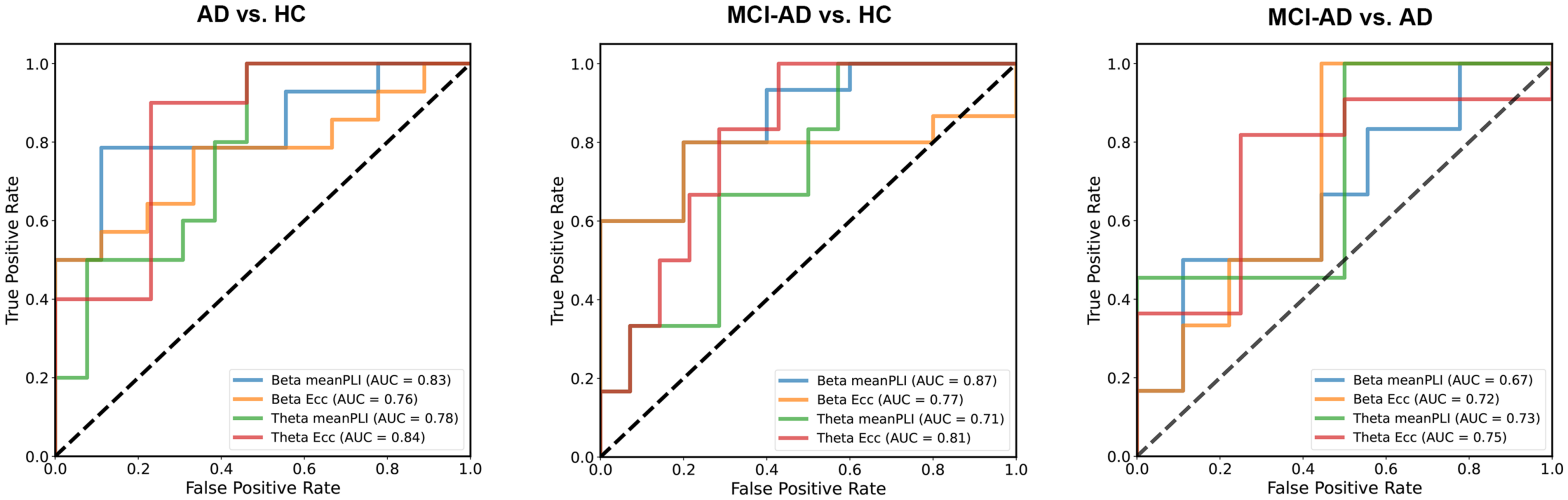
Classification of AD, MCI-AD, and HC using MST parameters. MST parameters from beta and theta band EEG recordings were evaluated for their ability to discriminate between AD, MCI, and HC groups using logistic regression with a 50% train-test split. For AD vs. HC, beta band mean PLI showed excellent performance (AUC = 0.83), with theta band eccentricity achieving the highest AUC (0.84). In distinguishing MCI-AD from HC, beta band mean PLI demonstrated the strongest discriminatory power (AUC = 0.87), followed by theta band eccentricity (AUC = 0.81). For MCI-AD vs. AD, theta band eccentricity showed moderate performance (AUC = 0.75), slightly outperforming mean PLI (AUC = 0.73).

In this study, we assessed the classification capabilities of specific MST parameters to differentiate AD, MCI-AD, and HC subjects using ROC curve analysis. We selected Eccentricity and mean PLI as the primary features for classification analysis based on their significant results in inter-group difference analysis. For distinguishing AD from HC, beta band mean PLI and theta band Eccentricity demonstrated excellent performance, with AUCs of 0.83 (95% CI: 0.675–1.0, *p* < 0.0001) and 0.84 (95% CI: 0.664–1.0, *p* < 0.0001), respectively. In differentiating MCI-AD from HC, beta band mean PLI showed the strongest discriminatory power (AUC = 0.87, 95% CI: 0.612–0.986, *p* = 0.0004), whereas theta band Eccentricity showed a trend but did not reach significance (AUC = 0.81, 95% CI: 0.457–0.941, *p* = 0.0608). When distinguishing MCI-AD from AD, theta band parameters exhibited moderate performance, with Eccentricity (AUC = 0.75, 95% CI: 0.491–1.0, *p* = 0.0151) slightly outperforming mean PLI (AUC = 0.73, 95% CI: 0.499–1.0, *p* = 0.0221). Notably, beta band parameters did not reach statistical significance in this comparison (Figure 6).

### 3.6 Associations between MST parameters and CSF biomarkers

**Figure 7:**
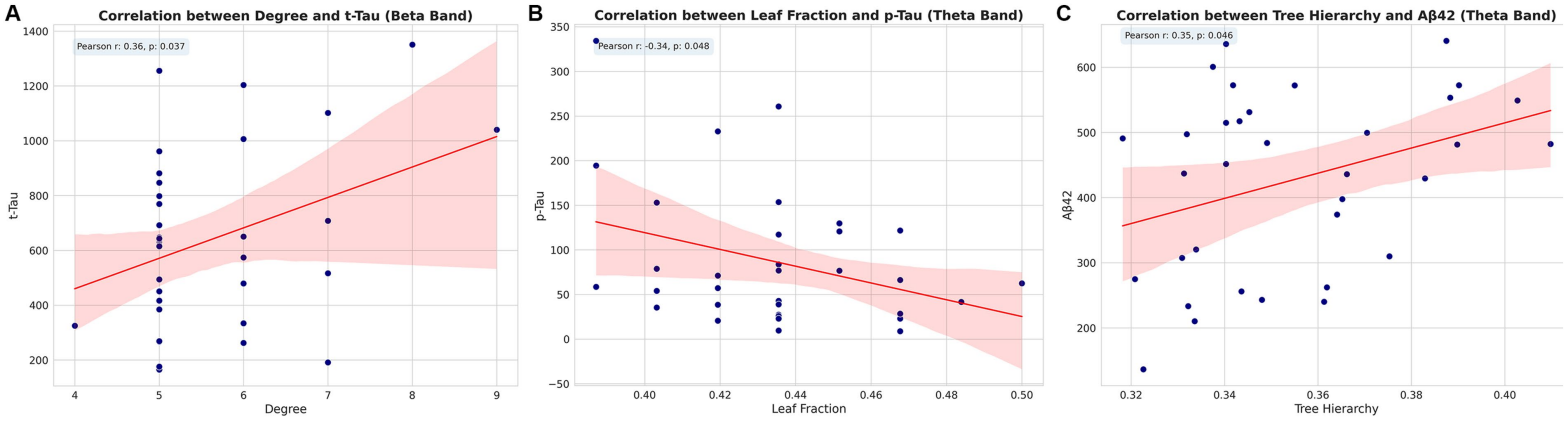
Correlations between MST parameters and CSF biomarkers. Scatter plots showing significant correlations between **(A)** degree and t-Tau in beta band, **(B)** leaf fraction and p-Tau in theta band, and **(C)** tree hierarchy and Aβ_1–42_ in theta band. Pearson correlation coefficients (r) and *p*-values are indicated for each plot. Pearson correlation coefficients (r) and *p*-values are indicated for each plot. t-Tau, total tau; p-Tau, phosphorylated tau.

Correlation analysis revealed significant associations between MST parameters and CSF biomarkers across different frequency bands, persisting after adjusting for sex, age, and years of education. In the beta band, degree exhibited a positive correlation with total tau (t-Tau) levels (Pearson r = 0.36, *p* = 0.037). Within the theta band, leaf fraction demonstrated a negative correlation with phosphorylated tau (p-Tau) levels (Pearson r = −0.34, *p* = 0.048), while tree hierarchy showed a positive correlation with Aβ_1–42_ levels (Pearson r = 0.35, *p* = 0.046). These findings indicate specific association patterns between MST parameters and AD-related CSF biomarkers across different frequency bands. The observed correlations, each unique to different MST parameters and CSF biomarkers, potentially reflect the complex relationship between brain network topology and AD pathology (Figure 7).

## 4 Discussion

This study examines functional brain network alterations across the AD spectrum using MST analysis of rsEEG data. We identified distinct frequency-specific changes in network topology that differentiate AD, MCI-AD, and healthy controls. Particularly notable were the increased connectivity and altered topology in the theta band and decreased connectivity in the beta band in AD patients, alongside unique network configurations in MCI-AD. Importantly, these network parameters correlated with cognitive performance and CSF biomarkers of AD pathology. Our analysis revealed that certain MST metric relationships in AD differ from theoretical expectations seen in healthy networks, suggesting that neurodegenerative processes fundamentally alter brain network organization principles. Rather than simple disruptions, these changes appear to represent specific pathological reorganization patterns that reflect the underlying disease process.

### 4.1 Frequency-specific network alterations

In the theta band, AD patients exhibited increased mean PLI, BC_max_, and Eccentricity compared to HC. The concurrent increases in BC_max_ (indicating higher centrality) and Eccentricity (indicating longer paths) challenge conventional network theory, which typically predicts an inverse relationship between these metrics. This unusual configuration suggests AD-specific network disruption where certain nodes become more critical for information flow while overall network integration decreases. This may result from neurodegenerative processes forcing communication through fewer surviving pathways while requiring longer routes between regions. While mean PLI provides valuable information about functional connectivity strength, it does not directly reflect how connections between nodes are organized. Our MST analysis addresses this limitation by examining topological properties independent of absolute connectivity values. This distinction between connectivity strength and network organization is crucial for interpreting the complex neural dynamics in AD. Conversely, in the beta band, we observed decreased mean PLI in AD patients, suggesting a more regularized network structure. These findings are consistent with previous studies on AD-related network changes. For instance, Stam et al. (2009) reported increased theta band functional connectivity in AD using synchronization likelihood. In contrast, Engels et al. (2015) found that AD patients had weaker functional connectivity than controls, especially in higher frequency bands, and that active regions seem more prone to AD pathology. Similarly, Koelewijn et al. (2017) observed disrupted alpha and beta-band resting-state oscillatory network connectivity in AD using MEG data. Notably, our study extends these findings by demonstrating specific changes in MST metrics, such as BC_max_ and Eccentricity, thereby providing a more detailed characterization of the topological alterations in both AD and MCI-AD. The posterior predominance observed in the theta band despite reduced global density may reflect compensatory recruitment in response to AD pathology, though this interpretation remains provisional and requires longitudinal confirmation.

These findings complement previous MST studies in neurodegenerative disorders, such as those by Yu et al. (2016) in frontotemporal dementia, Peraza et al. (2015) in dementia with Lewy bodies, and Ciftçi (2011) in Alzheimer’s disease. Our results align with the broader literature on network disruptions in neurodegenerative diseases, while providing novel insights into the frequency-specific alterations in AD and MCI-AD. For instance, van Dellen et al. (2015) reported a loss of EEG network efficiency in dementia with Lewy bodies, which was related to cognitive impairment. Additionally, Wang et al. (2014) found decreased coherence and functional connectivity in AD using traditional connectivity measures, further supporting the notion of network disruptions in neurodegenerative disorders. In the delta band, MCI-AD patients showed increased maximum degree despite a trend toward higher mean eccentricity, which appears contradictory as these metrics typically exhibit negative correlation. This pattern may reflect an early compensatory mechanism where specific nodes develop stronger centrality while overall network efficiency decreases, potentially characterizing the transitional state between healthy cognition and established AD. Intriguingly, the MCI-AD group did not display significant differences in mean PLI when compared to both the HC and AD groups in any frequency band. However, MST analysis revealed that in the beta band, the MCI-AD group had significantly different Kappa values compared to both AD and HC groups, suggesting a unique network topology in this prodromal stage of AD. The lower Kappa values in the MCI-AD group indicate a less uniform degree distribution, which may reflect early pathological processes such as synaptic dysfunction and neuronal loss (Tijms et al., 2013). Notably, the beta-band Kappa pattern in MCI-AD differed from both HC and AD, suggesting a non-linear, prodromal-specific reconfiguration rather than a simple intermediate state. This dissociation warrants emphasis as a candidate biomarker for the transitional phase; replication, test–retest reliability, and external validation are needed to establish clinical utility. This vulnerability in network structure may contribute to the cognitive deficits and increased risk of progression to AD observed in this population (Petersen et al., 2018). Furthermore, the specificity of these changes to the beta band highlights the importance of considering frequency-specific network alterations in the early stages of AD.

### 4.2 Network metrics and cognitive performance

In the theta band, cognitive scores negatively correlated with both BC_max_ and Eccentricity. Rather than being contradictory, these parallel correlations suggest that both increased centrality and increased path length represent maladaptive changes in AD. As cognitive function deteriorates, networks appear to develop greater dependence on central hubs alongside less efficient overall organization. This dual impairment may reflect increasingly constrained information flow as AD progresses. This is consistent with previous studies reporting increased theta band synchronization in AD and MCI patients (Koelewijn et al., 2017; Stam, 2014). The positive correlations between Leaf fraction and cognitive scores (HDS-R, AVLT-delay) further support this interpretation, suggesting that preserved network diversity relates to better cognitive performance, contrary to the conventional view that higher Leaf fraction necessarily indicates a less efficient network organization (Yu et al., 2016). In the beta band, cognitive scores positively correlated with mean PLI and Eccentricity. Notably, the positive correlation between diameter and cognitive scores requires careful interpretation, as increased diameter traditionally indicates less integrated topology. This counter-intuitive finding likely reflects compensatory mechanisms where the brain adapts to pathology through alternative communication pathways. In the beta band specifically, a degree of functional segregation may limit propagation of pathological synchronization while preserving essential cognitive processes. This observation aligns with previous studies reporting altered beta band synchronization in AD and MCI (Maestú et al., 2015; López et al., 2014), and suggests that conventional network efficiency metrics may require disease-specific interpretation. The positive correlations between Tree hierarchy and cognitive scores (MoCA, AVLT-delay) further suggest that certain aspects of network organization in the beta band contribute to maintaining cognitive function (Stam et al., 2007), though our interpretation emphasizes the potential adaptive value of maintained network differentiation rather than simply increased integration.

### 4.3 Diagnostic potential of MST parameters

The ROC analysis demonstrated the potential of MST parameters, particularly beta band mean PLI and theta band Eccentricity, in differentiating AD and MCI-AD from healthy controls. These findings are comparable to those reported by previous studies using MST measures to investigate brain network changes in AD. Canario et al. (2022) found that MST measures derived from functional near-infrared spectroscopy (fNIRS) data were effective in characterizing brain topologies and distinguishing AD patients from those with mild cognitive impairment (MCI) and healthy controls. Similarly, a systematic review by Blomsma et al. (2022) highlighted the potential of MST metrics for assessing disease specificity and transdiagnostic sensitivity in neurological and psychiatric conditions, including neurodegenerative disorders. However, our study extends this to include MCI-AD, highlighting the potential of these measures for early diagnosis. The moderate performance in distinguishing MCI-AD from AD suggests that while these measures are sensitive to early network changes, they may be less effective in tracking progression once significant pathology is established. This underscores the potential of MST parameters as early diagnostic biomarkers but also highlights the need for complementary measures to track disease progression. Clinically, this positions EEG-MST metrics primarily as tools for early detection and cohort enrichment, while progression monitoring in established AD may require composite models that combine MST features with longitudinal cognitive change, CSF/PET biomarkers, or structural/functional MRI to capture disease dynamics more sensitively.

### 4.4 CSF biomarkers and network topology

Our study is among the first to directly link MST parameters with CSF biomarkers of AD. The positive correlation between beta band Degree and t-Tau levels, and the negative correlation between theta band Leaf fraction and p-Tau levels, provide novel insights into the relationship between network topology and AD pathology. These findings extend previous work by Smailovic et al. (2021), who found that the relationships between EEG measures and CSF biomarkers are also reflected in the topological properties of functional networks, as well as the earlier work by Smailovic et al. (2018) on the correlation between quantitative EEG power/synchronization and Alzheimer’s disease CSF biomarkers. The positive correlation between theta band tree hierarchy and Aβ_1–42_ levels is noteworthy. Lower levels of Aβ_1–42_ in the CSF indicate more amyloid deposition in the brain, a hallmark of AD. This positive correlation suggests that more amyloid deposition (i.e., lower Aβ_1–42_ levels) is associated with a less complex or less ordered hierarchical structure in the theta band activity, possibly reflecting poorer cognitive function or more neurodegeneration. Interestingly, Palop and Mucke (2016), in their review of neural network dysfunction in AD, suggest that amyloid pathology may lead to network dysfunctions that precede overt neurodegeneration. Our finding of a less organized theta band hierarchy in the presence of more amyloid deposition aligns with this notion, potentially capturing an early stage of network disruption. From a neurobiological perspective, the observed frequency–biomarker mapping is plausible. Theta-band coordination is strongly shaped by hippocampo–thalamo–cortical loops and local inhibitory–excitatory balance; amyloid-related synaptic and interneuron dysfunction is therefore expected to preferentially perturb slower rhythms, which is consistent with a less organized theta hierarchy when amyloid burden is higher. By contrast, beta-band organization depends on long-range cortico–cortical coupling within frontoparietal and sensorimotor systems; axonal/myelin injury and neuronal loss associated with tau pathology could weaken distributed hub integration, in line with the beta-band topology changes and the Degree–t-Tau association. These mechanistic links are provisional and should be tested using multimodal or longitudinal designs (e.g., PET–EEG, source-level analyses).

Several methodological considerations warrant mention. First, we focused on MST topology rather than mean PLI alone, as MST provides specific insights into network organization. Second, the apparent contradictions between certain MST metrics likely reflect disease-specific reorganization patterns rather than methodological inconsistencies.

### 4.5 Limitation and future directions

Our study provides valuable insights into network alterations in AD and MCI-AD; however, several limitations should be acknowledged. Despite applying strict inclusion and exclusion criteria to ensure sample homogeneity and minimize confounding variables, our sample size, especially for the MCI-AD group, was relatively small, potentially limiting the generalizability of our findings. While we controlled for age, education, and handedness, other potential confounders such as medication use, comorbidities, and lifestyle factors were not accounted for, which may impact brain network organization. In particular, CNS-active medications and multimorbidity may systematically bias frequency-specific EEG features (e.g., sedative–hypnotics can increase beta activity, anticholinergic burden can alter slower rhythms), and we did not systematically record drug class, dose, or duration. Future studies should quantify medication exposure and perform sensitivity analyses to assess its impact on MST-derived metrics. Additionally, although we employed a comprehensive battery of clinical and neuropsychological assessments to evaluate participants’ cognitive function and emotional state, the cross-sectional design limits our ability to draw conclusions about the temporal dynamics of network changes throughout disease progression. Future studies with larger cohorts, consideration of a broader range of confounding factors, validation with alternative connectivity metrics like wPLI, and longitudinal designs are necessary to confirm our results, enhance generalizability, provide a more comprehensive understanding of network alterations in AD and MCI-AD, and elucidate how brain networks evolve as the disease progresses from the prodromal stage to full-blown AD. Finally, because MST-based topology relies on the rank ordering of edge weights, it emphasizes group-level connectivity patterns and may under-represent individual idiosyncrasies; subject-level reliability and individualized tree approaches were beyond our scope and warrant future work.

The potential of MST analysis in detecting network changes across the AD spectrum, as demonstrated in our study, underscores its utility as a valuable tool for investigating network alterations in neurodegenerative disorders. As discussed by Tewarie et al. (2015) and Stam et al. (2014), MST analysis provides an unbiased method for characterizing complex brain networks, making it a valuable tool for investigating network changes in AD. By employing this approach, we contribute to the growing body of evidence supporting the potential of network analysis techniques in uncovering early biomarkers of AD, as emphasized by Maestú et al. (2019) in their discussion of the potential of M/EEG in AD research.

## 5 Conclusion

In summary, our MST analysis revealed frequency-specific alterations in brain network topology across the AD spectrum. These changes correlated with cognitive performance and CSF biomarkers, while unusual relationships between MST metrics likely reflect disease-specific reorganization patterns. These findings underscore EEG-based MST analysis as a promising non-invasive tool for capturing AD network pathology. Future longitudinal studies are needed to track these network changes during disease progression.

## Data Availability

The raw data supporting the conclusions of this article will be made available by the authors, without undue reservation.

## References

[ref1] Albert M. S. DeKosky S. T. Dickson D. Dubois B. Feldman H. H. Fox N. C.. (2011). The diagnosis of mild cognitive impairment due to Alzheimer's disease: recommendations from the National Institute on Aging-Alzheimer's Association workgroups on diagnostic guidelines for Alzheimer's disease. Alzheimers Dement. 7, 270–279. doi: 10.1016/j.jalz.2011.03.008, PMID: 21514249 PMC3312027

[ref2] Babiloni C. Blinowska K. Bonanni L. Cichocki A. De Haan W. Del Percio C.. (2020). What electrophysiology tells us about Alzheimer's disease: a window into the synchronization and connectivity of brain neurons. Neurobiol. Aging 85, 58–73. doi: 10.1016/j.neurobiolaging.2019.09.008, PMID: 31739167

[ref3] Bahrami M. Laurienti P. J. Shappell H. M. Simpson S. L. (2023). Brain network analysis: a review on multivariate analytical methods. Brain Connect. 13, 64–79. doi: 10.1089/brain.2022.0007, PMID: 36006366 PMC10024592

[ref4] Becske M. Marosi C. Molnár H. Fodor Z. Farkas K. Rácz F. S.. (2024). Minimum spanning tree analysis of EEG resting-state functional networks in schizophrenia. Sci. Rep. 14:10495. doi: 10.1038/s41598-024-61316-8, PMID: 38714807 PMC11076461

[ref5] Benwell C. S. Y. Davila-Pérez P. Fried P. J. Jones R. N. Travison T. G. Santarnecchi E.. (2020). EEG spectral power abnormalities and their relationship with cognitive dysfunction in patients with Alzheimer's disease and type 2 diabetes. Neurobiol. Aging 85, 83–95. doi: 10.1016/j.neurobiolaging.2019.10.004, PMID: 31727363 PMC6942171

[ref6] Bijsterbosch J. Harrison S. J. Jbabdi S. Woolrich M. Beckmann C. Smith S.. (2020). Challenges and future directions for representations of functional brain organization. Nat. Neurosci. 23, 1484–1495. doi: 10.1038/s41593-020-00726-z, PMID: 33106677

[ref7] Blomsma N. de Rooy B. Gerritse F. van der Spek R. Tewarie P. Hillebrand A.. (2022). Minimum spanning tree analysis of brain networks: a systematic review of network size effects, sensitivity for neuropsychiatric pathology, and disorder specificity. Netw. Neurosci. 6, 301–319. doi: 10.1162/netn_a_00245, PMID: 35733422 PMC9207994

[ref8] Canario E. Chen D. Han Y. Niu H. Biswal B. (2022). Global network analysis of Alzheimer’s disease with minimum spanning trees. J Alzheimer's Dis 89, 571–581. doi: 10.3233/JAD-215573, PMID: 35938244 PMC13130941

[ref9] Ciftçi K. (2011). Minimum spanning tree reflects the alterations of the default mode network during Alzheimer's disease. Ann. Biomed. Eng. 39, 1493–1504. doi: 10.1007/s10439-011-0258-9, PMID: 21286814

[ref10] Crimi A. Giancardo L. Sambataro F. Gozzi A. Murino V. Sona D. (2019). MultiLink analysis: brain network comparison via sparse connectivity analysis. Sci. Rep. 9:65. doi: 10.1038/s41598-018-37300-4, PMID: 30635604 PMC6329758

[ref11] Davatzikos C. Bhatt P. Shaw L. M. Batmanghelich K. N. Trojanowski J. Q. (2011). Prediction of MCI to AD conversion, via MRI, CSF biomarkers, and pattern classification. Neurobiol. Aging 32, 2322.e19–2322.e27. doi: 10.1016/j.neurobiolaging.2010.05.023, PMID: 20594615 PMC2951483

[ref12] Delorme A. Makeig S. (2004). EEGLAB: an open source toolbox for analysis of single-trial EEG dynamics including independent component analysis. J. Neurosci. Methods 134, 9–21. doi: 10.1016/j.jneumeth.2003.10.009, PMID: 15102499

[ref13] Engels M. M. A. Stam C. J. van der Flier W. M. Scheltens P. de Waal H. van Straaten E. C. W. (2015). Declining functional connectivity and changing hub locations in Alzheimer’s disease: an EEG study. BMC Neurol. 15:145. doi: 10.1186/s12883-015-0400-7, PMID: 26289045 PMC4545875

[ref14] Engels M. M. A. van der Flier W. M. Stam C. J. Hillebrand A. Scheltens P. van Straaten E. C. W. (2017). Alzheimer's disease: the state of the art in resting-state magnetoencephalography. Clin. Neurophysiol. 128, 1426–1437. doi: 10.1016/j.clinph.2017.05.012, PMID: 28622527

[ref15] Gaugler J. James B. Johnson T. Reimer J. Solis M. Weuve J.. (2022). Alzheimer's disease facts and figures. Alzheimers Dement. 18, 700–789. doi: 10.1002/alz.12638 35289055

[ref16] Horton D. K. Hynan L. S. Lacritz L. H. Rossetti H. C. Weiner M. F. Cullum C. M. (2015). An abbreviated Montreal cognitive assessment (MoCA) for dementia screening. Clin. Neuropsychol. 29, 413–425. doi: 10.1080/13854046.2015.1043349, PMID: 25978540 PMC4501880

[ref17] Horvath A. Szucs A. Csukly G. Sakovics A. Stefanics G. Kamondi A. (2018). EEG and ERP biomarkers of Alzheimer's disease: a critical review. Front. Biosci. (Landmark Ed) 23, 183–220. doi: 10.2741/4587, PMID: 28930543

[ref18] Imai Y. Hasegawa K. (1994). The revised Hasegawa's dementia scale (HDS-R)-evaluation of its usefulness as a screening test for dementia. J. Hong Kong Coll. Psychiatr. 4, 20–24.

[ref19] Jack C. R. Jr. Bennett D. A. Blennow K. Carrillo M. C. Dunn B. Haeberlein S. B.. (2018). Dementia, NIA-AA research framework: toward a biological definition of Alzheimer's disease. Alzheimers Dement. 14, 535–562. doi: 10.1016/j.jalz.2018.02.018. 29653606 PMC5958625

[ref20] Kehm C. S. Fassbender R. V. Onur O. A. (2023). Functional connectivity in Alzheimer’s disease: a resting-state EEG study. Alzheimers Dement. 19:e074707. doi: 10.1002/alz.074707

[ref21] Koelewijn L. Bompas A. Tales A. Brookes M. J. Muthukumaraswamy S. D. Bayer A.. (2017). Alzheimer's disease disrupts alpha and beta-band resting-state oscillatory network connectivity. Clin. Neurophysiol. 128, 2347–2357. doi: 10.1016/j.clinph.2017.04.018, PMID: 28571910 PMC5674981

[ref22] Kruskal J. B. (1956). On the shortest spanning subtree of a graph and the traveling salesman problem. Proc. Am. Math. Soc. 7, 48–50. doi: 10.1090/S0002-9939-1956-0078686-7

[ref23] López M. E. Bruña R. Aurtenetxe S. Pineda-Pardo J. Marcos A. Arrazola J.. (2014). Alpha-band hypersynchronization in progressive mild cognitive impairment: a magnetoencephalography study. J. Neurosci. 34, 14551–14559. doi: 10.1523/jneurosci.0964-14.2014 25355209 PMC6608420

[ref24] Maestú F. Cuesta P. Hasan O. Fernandéz A. Funke M. Schulz P. E. (2019). The importance of the validation of M/EEG with current biomarkers in Alzheimer's disease. Front. Hum. Neurosci. 13:17. doi: 10.3389/fnhum.2019.00017, PMID: 30792632 PMC6374629

[ref25] Maestú F. Peña J. M. Garcés P. González S. Bajo R. Bagic A.. (2015). A multicenter study of the early detection of synaptic dysfunction in mild cognitive impairment using magnetoencephalography-derived functional connectivity. Neuroimage Clin. 9, 103–109. doi: 10.1016/j.nicl.2015.07.011 26448910 PMC4552812

[ref26] Maier W. Buller R. Philipp M. Heuser I. (1988). The Hamilton anxiety scale: reliability, validity and sensitivity to change in anxiety and depressive disorders. J. Affect. Disord. 14, 61–68. doi: 10.1016/0165-0327(88)90072-9, PMID: 2963053

[ref27] Meghdadi A. H. Karic M. S. Richard C. Waninger S. Mcconnell M. Poole J.. (2021). EEG biomarkers differentiate Lewy body dementia from Alzheimer’s disease. Alzheimers Dement. 17:e051386. doi: 10.1002/alz.051386

[ref28] Morris J. C. (1993). The clinical dementia rating (CDR): current version and scoring rules. Neurology 43, 2412–2414. doi: 10.1212/wnl.43.11.2412-a 8232972

[ref29] Mulder C. Verwey N. A. van der Flier W. M. Bouwman F. H. Kok A. van Elk E. J.. (2010). Amyloid-beta(1-42), total tau, and phosphorylated tau as cerebrospinal fluid biomarkers for the diagnosis of Alzheimer disease. Clin. Chem. 56, 248–253. doi: 10.1373/clinchem.2009.130518 19833838

[ref30] Nichols E. Steinmetz J. D. Vollset S. E. Fukutaki K. Chalek J. Abd-Allah F.. (2022). Estimation of the global prevalence of dementia in 2019 and forecasted prevalence in 2050: an analysis for the global burden of disease study 2019. Lancet Public Health 7, e105–e125. doi: 10.1016/S2468-2667(21)00249-8. 34998485 PMC8810394

[ref31] O'Hara M. W. Rehm L. P. (1983). Hamilton rating scale for depression: reliability and validity of judgments of novice raters. J. Consult. Clin. Psychol. 51, 318–319. doi: 10.1037//0022-006x.51.2.318 6841779

[ref32] Oostenveld R. Fries P. Maris E. Schoffelen J. M. (2011). FieldTrip: open source software for advanced analysis of MEG, EEG, and invasive electrophysiological data. Comput. Intell. Neurosci. 2011:156869. doi: 10.1155/2011/156869, PMID: 21253357 PMC3021840

[ref33] Palop J. J. Mucke L. (2016). Network abnormalities and interneuron dysfunction in Alzheimer disease. Nat. Rev. Neurosci. 17, 777–792. doi: 10.1038/nrn.2016.141, PMID: 27829687 PMC8162106

[ref34] Peraza L. R. Taylor J. P. Kaiser M. (2015). Divergent brain functional network alterations in dementia with Lewy bodies and Alzheimer's disease. Neurobiol. Aging 36, 2458–2467. doi: 10.1016/j.neurobiolaging.2015.05.015, PMID: 26115566 PMC4706129

[ref35] Perez-Valero E. Lopez-Gordo M. A. Morillas C. Pelayo F. Vaquero-Blasco M. A. (2021). A review of automated techniques for assisting the early detection of Alzheimer's disease with a focus on EEG. J Alzheimer's Dis 80, 1363–1376. doi: 10.3233/JAD-201455, PMID: 33682717

[ref36] Petersen R. C. Lopez O. Armstrong M. J. Getchius T. S. D. Ganguli M. Gloss D.. (2018). Practice guideline update summary: mild cognitive impairment: report of the guideline development, dissemination, and implementation subcommittee of the American Academy of Neurology. Neurology 90, 126–135. doi: 10.1212/wnl.0000000000004826 29282327 PMC5772157

[ref37] Reijneveld J. C. Ponten S. C. Berendse H. W. Stam C. J. (2007). The application of graph theoretical analysis to complex networks in the brain. Clin. Neurophysiol. 118, 2317–2331. doi: 10.1016/j.clinph.2007.08.010, PMID: 17900977

[ref38] Scheijbeler E. P. de Haan W. Stam C. J. Twisk J. W. R. Gouw A. A. (2023). Longitudinal resting-state EEG in amyloid-positive patients along the Alzheimer's disease continuum: considerations for clinical trials. Alzheimer's Res Ther 15:182. doi: 10.1186/s13195-023-01327-1, PMID: 37858173 PMC10585755

[ref39] Scheltens P. De Strooper B. Kivipelto M. Holstege H. Chételat G. Teunissen C. E.. (2021). Alzheimer's disease. Lancet 397, 1577–1590. doi: 10.1016/S0140-6736(20)32205-4, PMID: 33667416 PMC8354300

[ref40] Smailovic U. Kåreholt I. Koenig T. Ashton N. J. Winblad B. Höglund K.. (2021). Synaptic molecular and neurophysiological markers are independent predictors of progression in Alzheimer's disease. J Alzheimer's Dis 83, 355–366. doi: 10.3233/jad-201234, PMID: 34334389 PMC8461684

[ref41] Smailovic U. Koenig T. Kåreholt I. Andersson T. Kramberger M. G. Winblad B.. (2018). Quantitative EEG power and synchronization correlate with Alzheimer's disease CSF biomarkers. Neurobiol. Aging 63, 88–95. doi: 10.1016/j.neurobiolaging.2017.11.005, PMID: 29245058

[ref42] Song J. Birn R. M. Boly M. Meier T. B. Nair V. A. Meyerand M. E.. (2014). Age-related reorganizational changes in modularity and functional connectivity of human brain networks. Brain Connect. 4, 662–676. doi: 10.1089/brain.2014.0286, PMID: 25183440 PMC4238253

[ref43] Stam C. J. (2014). Modern network science of neurological disorders. Nat. Rev. Neurosci. 15, 683–695. doi: 10.1038/nrn3801, PMID: 25186238

[ref44] Stam C. J. de Haan W. Daffertshofer A. Jones B. F. Manshanden I. van Cappellen A. M.. (2009). Graph theoretical analysis of magnetoencephalographic functional connectivity in Alzheimer's disease. Brain 132, 213–224. doi: 10.1093/brain/awn262, PMID: 18952674

[ref45] Stam C. J. Jones B. F. Nolte G. Breakspear M. Scheltens P. (2007). Small-world networks and functional connectivity in Alzheimer's disease. Cereb. Cortex 17, 92–99. doi: 10.1093/cercor/bhj127, PMID: 16452642

[ref46] Stam C. J. Nolte G. Daffertshofer A. (2007). Phase lag index: assessment of functional connectivity from multi channel EEG and MEG with diminished bias from common sources. Hum. Brain Mapp. 28, 1178–1193. doi: 10.1002/hbm.20346, PMID: 17266107 PMC6871367

[ref47] Stam C. J. Tewarie P. Van Dellen E. van Straaten E. C. Hillebrand A. Van Mieghem P. (2014). The trees and the forest: characterization of complex brain networks with minimum spanning trees. Int. J. Psychophysiol. 92, 129–138. doi: 10.1016/j.ijpsycho.2014.04.001 24726900

[ref48] Tait L. Stothart G. Coulthard E. Brown J. T. Kazanina N. Goodfellow M. (2019). Network substrates of cognitive impairment in Alzheimer's disease. Clin. Neurophysiol. 130, 1581–1595. doi: 10.1016/j.clinph.2019.05.027, PMID: 31306967

[ref49] Tewarie P. Hillebrand A. Schoonheim M. M. van Dijk B. W. Geurts J. J. G. Barkhof F.. (2014). Functional brain network analysis using minimum spanning trees in multiple sclerosis: an MEG source-space study. NeuroImage 88, 308–318. doi: 10.1016/j.neuroimage.2013.10.022, PMID: 24161625

[ref50] Tewarie P. van Dellen E. Hillebrand A. Stam C. J. (2015). The minimum spanning tree: an unbiased method for brain network analysis. NeuroImage 104, 177–188. doi: 10.1016/j.neuroimage.2014.10.015, PMID: 25451472

[ref51] Tijms B. M. Wink A. M. de Haan W. van der Flier W. M. Stam C. J. Scheltens P.. (2013). Alzheimer's disease: connecting findings from graph theoretical studies of brain networks. Neurobiol. Aging 34, 2023–2036. doi: 10.1016/j.neurobiolaging.2013.02.020, PMID: 23541878

[ref52] Tombaugh T. N. McIntyre N. J. (1992). The Mini-mental state examination: a comprehensive review. J. Am. Geriatr. Soc. 40, 922–935. doi: 10.1111/j.1532-5415.1992.tb01992.x, PMID: 1512391

[ref53] Vakil E. Blachstein H. (1993). Rey auditory-verbal learning test: structure analysis. J. Clin. Psychol. 49, 883–890. doi: 10.1002/1097-4679(199311)49:6<883::AID-JCLP2270490616>3.0.CO;2-6, PMID: 8300877

[ref54] van Dellen E. de Waal H. van der Flier W. M. Lemstra A. W. Slooter A. J. Smits L. L.. (2015). Loss of EEG network efficiency is related to cognitive impairment in dementia with Lewy bodies. Mov. Disord. 30, 1785–1793. doi: 10.1002/mds.26309, PMID: 26179663

[ref55] van Dellen E. Douw L. Hillebrand A. de Witt Hamer P. C. Baayen J. C. Heimans J. J.. (2014). Epilepsy surgery outcome and functional network alterations in longitudinal MEG: a minimum spanning tree analysis. NeuroImage 86, 354–363. doi: 10.1016/j.neuroimage.2013.10.010, PMID: 24128736

[ref56] van Lutterveld R. van Dellen E. Pal P. Yang H. Stam C. J. Brewer J. (2017). Meditation is associated with increased brain network integration. NeuroImage 158, 18–25. doi: 10.1016/j.neuroimage.2017.06.071, PMID: 28663069 PMC5614811

[ref57] Wang R. Wang J. Yu H. Wei X. Yang C. Deng B. (2014). Decreased coherence and functional connectivity of electroencephalograph in Alzheimer's disease. Chaos 24:033136. doi: 10.1063/1.4896095, PMID: 25273216

[ref58] Youssef N. Xiao S. Liu M. Lian H. Li R. Chen X.. (2021). Functional brain networks in mild cognitive impairment based on resting electroencephalography signals. Front. Comput. Neurosci. 15:698386. doi: 10.3389/fncom.2021.698386, PMID: 34776913 PMC8579961

[ref59] Yu M. Gouw A. A. Hillebrand A. Tijms B. M. Stam C. J. van Straaten E. C.. (2016). Different functional connectivity and network topology in behavioral variant of frontotemporal dementia and Alzheimer's disease: an EEG study. Neurobiol. Aging 42, 150–162. doi: 10.1016/j.neurobiolaging.2016.03.018, PMID: 27143432

